# Olfactory consciousness and gamma oscillation couplings across the olfactory bulb, olfactory cortex, and orbitofrontal cortex

**DOI:** 10.3389/fpsyg.2013.00743

**Published:** 2013-10-16

**Authors:** Kensaku Mori, Hiroyuki Manabe, Kimiya Narikiyo, Naomi Onisawa

**Affiliations:** ^1^Department of Physiology, Graduate School of Medicine, The University of TokyoTokyo, Japan; ^2^Japan Science and Technology AgencyCREST, Tokyo, Japan

**Keywords:** olfactory cortex, orbitofrontal cortex, tufted and mitral cells, olfactory bulb, olfactory consciousness, gamma synchronization

## Abstract

The orbitofrontal cortex receives multi-modality sensory inputs, including olfactory input, and is thought to be involved in conscious perception of the olfactory image of objects. Generation of olfactory consciousness may require neuronal circuit mechanisms for the “binding” of distributed neuronal activities, with each constituent neuron representing a specific component of an olfactory percept. The shortest neuronal pathway for odor signals to reach the orbitofrontal cortex is olfactory sensory neuron—olfactory bulb—olfactory cortex—orbitofrontal cortex, but other pathways exist, including transthalamic pathways. Here, we review studies on the structural organization and functional properties of the shortest pathway, and propose a model of neuronal circuit mechanisms underlying the temporal bindings of distributed neuronal activities in the olfactory cortex. We describe a hypothesis that suggests functional roles of gamma oscillations in the bindings. This hypothesis proposes that two types of projection neurons in the olfactory bulb, tufted cells and mitral cells, play distinct functional roles in bindings at neuronal circuits in the olfactory cortex: tufted cells provide specificity-projecting circuits which send odor information with early-onset fast gamma synchronization, while mitral cells give rise to dispersedly-projecting feed-forward binding circuits which transmit the response synchronization timing with later-onset slow gamma synchronization. This hypothesis also suggests a sequence of bindings in the olfactory cortex: a small-scale binding by the early-phase fast gamma synchrony of tufted cell inputs followed by a larger-scale binding due to the later-onset slow gamma synchrony of mitral cell inputs. We discuss that behavioral state, including wakefulness and sleep, regulates gamma oscillation couplings across the olfactory bulb, olfactory cortex, and orbitofrontal cortex.

## Introduction

The human brain has the faculty of conscious olfactory perception. The orbitofrontal cortex (OFC) is thought to play a critical role in the generation of olfactory consciousness (Plailly et al., 2008; Li et al., 2010). In the olfactory pathway of primates, including humans, the OFC is the first neocortical stage that integrates multi-sensory modalities, including olfaction and taste, and computes reward value of the multi-sensory information to adaptively modify behavioral output (Rolls and Baylis, 1994; Rolls, 2006; Gottfried, 2007; Price, 2007; Wallis, 2007). Particularly, neuronal circuits in the human OFC are thought to be involved in the conscious perception of food flavor that requires integration of not only olfactory and taste signals but also virtually all sensory modalities (Rolls, 2006; Shepherd, 2006, 2007). A key feature of the conscious perception in general is that all of the multi-modality sensory components of objects are integrated as a unified whole (Tononi and Edelman, 1998; Crick and Koch, 2005).

Rodent OFC is also a key player in multi-modality integration and the reward valuation of sensory information for the flexibility of associative encoding (Schoenbaum et al., 2007). Although it is controversial whether rodents have the faculty of conscious olfactory perception and there is considerable anatomical variation in the organization of olfactory OFC across mammalian species (Gottfried, 2007; Price, 2007), we argue that understanding the neuronal circuit mechanisms that allow external odor information to become available to the rodent OFC during alert wakefulness will substantially contribute to understanding the neuronal circuit mechanisms which underlie human olfactory consciousness.

Here, we initially review recent advances in knowledge of the structural architecture and functional properties of the neuronal pathways that convey the odor information detected by sensory neurons in the nose to the OFC of rodents. We address the question of how and in which timing during a single inhalation-exhalation sniff cycle the external odor information is transmitted via the neuronal circuits of the olfactory bulb and piriform cortex to the OFC. We then propose the hypothesis that gamma oscillatory inputs from tufted cell and mitral cell axons play distinct functional roles in processing odor information in the piriform cortex and OFC.

**Figure 1:**
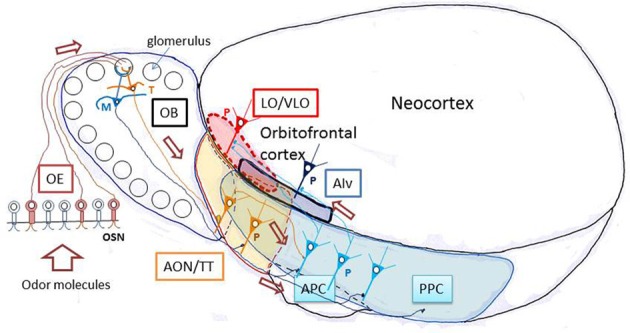
**Olfactory pathways to the orbitofrontal cortex in rodents.** A schematic diagram of the lateral view of the rodent brain illustrating the neuronal pathways from olfactory sensory neurons through the olfactory bulb and olfactory cortex to the orbitofrontal cortex. AIv, ventral agranular insular cortex; AON, anterior olfactory nucleus; APC, anterior piriform cortex; LO, lateral orbital cortex; M, mitral cell; OB, olfactory bulb; OE, olfactory epithelium; OSN, olfactory sensory neuron; P, pyramidal cell; PPC, posterior piriform cortex; T, tufted cell; TT, tenia tecta; VLO, ventrolateral orbital cortex. The orbitofrontal cortex (LO/VLO) is surrounded by red broken line.

As shown in Figure 1, odor molecules are received by olfactory sensory neurons in the olfactory epithelium. Olfactory sensory neurons send the odor information via their axons to the olfactory bulb, the first information processing center in the olfactory system (Mori et al., 1999; Shepherd et al., 2004). Principal neurons in the olfactory bulb, namely tufted cells and mitral cells, receive excitatory synaptic input from olfactory sensory neurons and send the signal directly or indirectly via olfactory peduncle areas to the pyramidal cells in the piriform cortex, a phylogenetically old cortex (paleocortex) with a relatively simple three-layered structure (Neville and Haberly, 2004). The odor signal is processed in the piriform cortex and then sent to higher olfactory association regions such as the amygdala, olfactory tubercle, and OFC. Pyramidal cells in the anterior piriform cortex (APC) project axons directly to the OFC, the first stage of the olfactory system in the neocortex. In the shortest pathway, therefore, the OFC is only three synapses distant from the olfactory sensory neurons. This characteristic short pathway from olfactory sensory neurons to the OFC is in a striking contrast with other sensory modalities, such as the visual and auditory systems, which involve many more relay stages before the sensory information reaches the prefrontal cortex. The short pathway of the olfactory system makes it an excellent and simple model system with which to analyze neuronal circuit mechanisms for the transfer of sensory information from the sensory neurons to the prefrontal cortex in the mammalian brain (Shepherd, 2007).

In the neocortex, thalamus, and hippocampus, synchronized spike discharges of large ensembles of neurons occur at gamma-range frequency (30–100 Hz) during alert wakefulness, and are thought to be associated with the spatial “binding” of distributed components of a sensory percept (Singer, 1999; Engel et al., 2001; Fries, 2009; Buzsaki and Wang, 2012). Behavioral state, including wakefulness and sleep, regulates the generation of gamma oscillations. In addition, gamma oscillation coupling is widely observed in the neuronal pathways of the mammalian brain and is an efficient way of transmitting information from one region to its target regions (Buzsaki and Wang, 2012). Gamma oscillations in thalamocortical networks during alert wakefulness are thought to be associated with the global “binding” of distributed aspects of a sensory percept (Gray et al., 1989; Singer and Gray, 1995; Singer, 1998).

Gamma oscillations of local field potentials in the olfactory bulb and olfactory cortex have been studied extensively (Adrian, 1942; Freeman, 1975; Mori and Takagi, 1977; Bressler, 1984; Buonviso et al., 2003; Friedman and Strowbridge, 2003; Kay, 2003; Neville and Haberly, 2003, 2004; Cenier et al., 2008; Rosero and Aylwin, 2011). The rodent OFC also shows gamma oscillations during olfaction-dependent tasks (van Wingerden et al., 2010), suggesting that gamma oscillatory activity of the central olfactory system plays a functional role in conveying odor signals from the olfactory bulb to the OFC via the APC. In the present study, we focus on the functional roles of odor inhalation-induced gamma oscillations in the olfactory bulb, APC and OFC, and discuss the possibility that behavioral state regulates gamma oscillation coupling across these areas in such a way that the transfer of odor information to the OFC is greatly facilitated when rodents are awake and paying attention to the odor information, but is diminished during sleep. We also discuss the occurrence of gamma oscillation couplings across the OFC, olfactory cortex and olfactory bulb during the exhalation phase of a respiratory cycle, the phase in which the brain is isolated from external odor information but receives strong retronasal stimulation from internal odors that originate from foods within the mouth.

## Neuronal pathways from olfactory sensory neurons to the orbitofrontal cortex

Before discussing the possible manner of odor information transmission along the olfactory pathways to the OFC, we will briefly review the characteristic structural organization of the central olfactory system, starting from the olfactory sensory neurons in the olfactory epithelium (Figure 1). Odor molecules are received by odorant receptors expressed on the cilial surface membrane of olfactory sensory neurons (Buck and Axel, 1991). Approximately 1000 different odorant receptor species occur in the mouse, and each olfactory sensory neuron expresses only one functional odorant receptor gene (one cell—one receptor rule). Each olfactory sensory neuron projects a single axon (olfactory axon) to a single glomerulus in the olfactory bulb, which has ~1800 glomeruli spatially arranged around its surface. Axons of numerous olfactory sensory neurons expressing the same type of odorant receptor converge onto two target glomeruli located at fixed positions in the olfactory bulb. Each glomerulus therefore represents a single type of odorant receptor (one glomerulus—one receptor rule) and detects specific “molecular features” of odorants (Mori et al., 1992, 1999). The spatial arrangement of glomeruli thus forms odorant receptor maps at the surface of the olfactory bulb (Mori et al., 2006; Mori and Sakano, 2011).

Within each glomerulus of the olfactory bulb, olfactory axons form excitatory synaptic connections on the terminal tuft of primary dendrites of two types of projection neurons, tufted cells, and mitral cells (Figure 1). Because of the one glomerulus—one receptor rule, all the olfactory axons and all the tufted and mitral cells that project to a single glomerulus form a functional module (glomerular module or glomerular unit) that represents a single type of odorant receptor (Shepherd et al., 2004; Mori and Sakano, 2011).

Individual mitral cells have their large cell bodies in the mitral cell layer and send dispersedly-projecting axons to virtually all areas of the olfactory cortex, including the piriform cortex (APC and posterior piriform cortex), areas in the olfactory peduncle [anterior olfactory nucleus (AON), tenia tecta, and dorsal peduncular cortex], olfactory tubercle, cortical amygdaloid nuclei, and lateral entorhinal cortex (Haberly and Price, 1977; Luskin and Price, 1983; Shipley and Ennis, 1996; Neville and Haberly, 2004; Igarashi et al., 2012). Tufted cells have smaller cell bodies that are distributed in the external plexiform layer (EPL). Each tufted cell projects axons selectively to focal targets in the olfactory peduncle areas (AON and tenia tecta) and the rostroventral part of the APC (Igarashi et al., 2012). Pyramidal cells in the AON give rise to associational axons that terminate in the piriform cortex.

**Figure 2:**
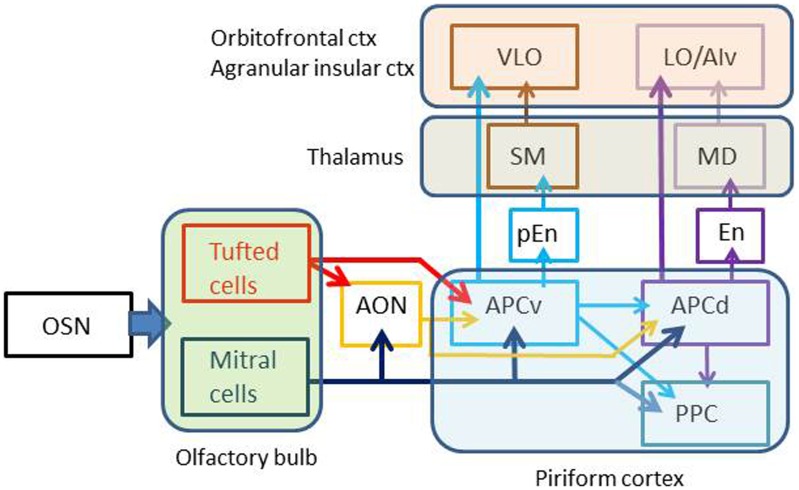
**Multiple parallel pathways from the olfactory sensory neurons to the orbitofrontal cortex.** Arrows indicate axonal projection. AON, anterior olfactory nucleus; APCv, ventral part of the anterior piriform cortex; APCd, dorsal part of the anterior piriform cortex; PPC, posterior piriform cortex; pEn pre-endopiriform nucleus; En, endopiriform nucleus; SM, submedius nucleus of thalamus; MD, mediodorsal nucleus of thalamus; OSN, olfactory sensory neuron; VLO, ventrolateral orbital cortex; LO, lateral orbital cortex; AIv, ventral agranular insular cortex. Olfactory bulb gives rise to parallel tufted and mitral cell pathways to the olfactory cortex. APCv sends odor information directly to the VLO or indirectly via the pEn and SM, while APCd transfers odor information directly to the LO/AIv or indirectly via the En and MD.

Pyramidal cells in layer II of the APC project axons to the OFC and ventral agranular insular cortex (AIv) of the neocortex. The projection from the APC to the OFC/AIv is composed of two parallel pathways (Figure 2) (Ekstrand et al., 2001). Pyramidal cells in the ventral part of the APC (APCv) project axons to the ventrolateral orbital cortex (VLO), while those in the dorsal APC (APCd) send axons to the lateral orbital cortex (LO) and AIv.

In addition to direct projection from the APC to OFC/AIv, indirect transthalamic pathways are also present. The APCd projects axons to the endopiriform nucleus (En), which projects axons to the mediodorsal nucleus (MD) of the thalamus (Figure 2). Thalamocortical neurons in the MD project to the LO and AIv. The APCv connects with the pre-endopiriform nucleus (pEn), which projects axons to the submedius nucleus (SM) of the thalamus. Thalamocortical neurons in the SM send axons to the VLO.

## Fast and slow gamma oscillations in the olfactory bulb: relation to tufted cell and mitral cell circuits

As early as 1942; Adrian reported prominent gamma range oscillatory activity in the olfactory bulb (Adrian, 1942). Since then a number of studies have shown that odor inhalation induces gamma oscillations of local field potentials (LFPs) in the olfactory bulb (Freeman, 1975; Bressler, 1984; Buonviso et al., 2003; Neville and Haberly, 2003; Cenier et al., 2008; Rosero and Aylwin, 2011). The gamma oscillations reflect synchronized spike discharges of tufted cells and mitral cells (Kashiwadani et al., 1999). Dendrodendritic reciprocal synaptic interactions between the excitatory projection neurons (tufted and mitral cells) and inhibitory interneurons (granule cells) participate in the generation of gamma oscillations in the olfactory bulb (Rall and Shepherd, 1968; Mori and Takagi, 1977; Friedman and Strowbridge, 2003; Lagier et al., 2004; Shepherd et al., 2004).

**Figure 3:**
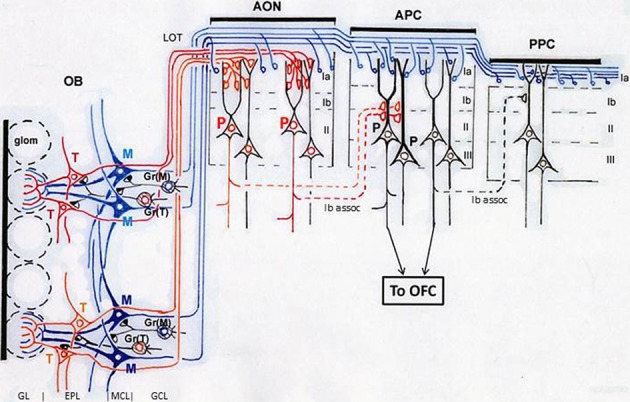
**Structural organization of tufted cell circuits and mitral cell circuits in the olfactory bulb and olfactory cortex.** In the olfactory bulb (OB), tufted cells (red and orange T) extend relatively short lateral dendrites in the superficial sub-lamina of the EPL and make dendrodendritic reciprocal synaptic connections mainly with tufted cell-targeting granule cells [Gr(T)]. Mitral cells (blue M) extend long lateral dendrites in the deep sub-lamina of the EPL and form dendrodendritic synapses mainly with mitral cell-targeting granule cells [Gr(M)]. Tufted cells project axons (red and orange lines) to focal targets in the olfactory peduncle areas including AON. Mitral cells project axons (blue lines) dispersedly to nearly all areas of the olfactory cortex. Layers of the olfactory bulb: GL, glomerular layer; EPL, external plexiform layer; MCL, mitral cell layer; GCL, granule cell layer. Layers in the olfactory cortex: Ia, layer Ia; Ib, layer Ib; II, layer II; III, layer III. LOT, lateral olfactory tract; Ib assoc, Ib associational axon. Red P indicates pyramidal cells in the AON. Black P shows pyramidal cells in the APC. Glom, glomerulus; OFC, orbitofrontal cortex.

As indicated in Figure 3, mitral cells (blue) project long lateral dendrites in the deeper sub-lamina of the external plexiform layer (EPL) and have dendrodendritic reciprocal synaptic interactions in the sub-lamina preferentially with mitral cell-targeting granule cells [Gr(M)], forming a mitral cell circuit (Mori et al., 1983; Orona et al., 1983; Greer, 1987). In contrast, tufted cells (red) extend relatively short lateral dendrites in the superficial sub-lamina of the EPL and have dendrodendritic synaptic interactions mainly with tufted cell-targeting granule cells [Gr(T)], forming a tufted cell circuit. These results raise the possibility that the odor inhalation-induced gamma oscillations encompass two distinct gamma oscillatory sources, a mitral cell circuit, and a tufted cell circuit.

**Figure 4:**
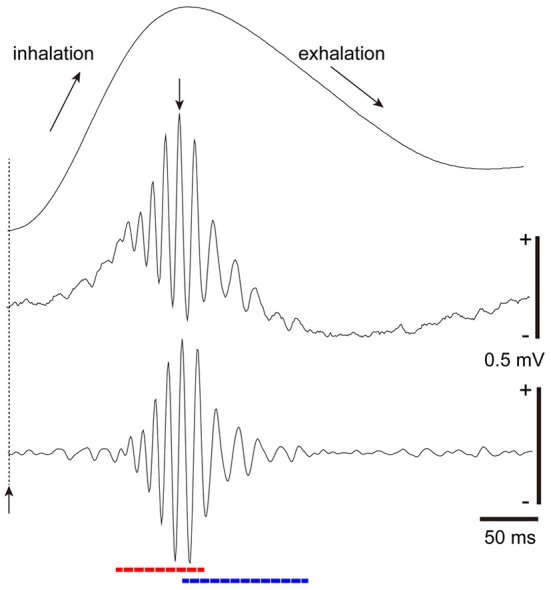
**Fast and slow gamma oscillations in the olfactory bulb.** Middle trace: sniff rhythm-paced gamma oscillations recorded from the granule cell layer of the olfactory bulb of a freely behaving rat. The sniff-induced local field potentials (LFPs) were averaged (*n* = 277 sniffs) in reference to the peak (a downward arrow) of gamma oscillations that occur near the phase of transition from inhalation to exhalation. Bottom trace: the local field potential shown in the middle trace was band-path filtered between 30 and 140 Hz. The sniff-paced gamma oscillations consist of early-onset fast gamma oscillation (red dashed line) and later-onset slow gamma oscillation (blue dashed line). Uppermost trace: respiration monitor via a thermocouple implanted in the nasal cavity. Upward reflection indicates inhalation. Sniff-onset is indicated by a vertical broken line with an upward arrow.

From this notion, we studied the temporal structure of the gamma oscillations and spike activity of tufted cells and mitral cells during a single inhalation-exhalation sniff cycle. As exemplified in Figure 4, simultaneous recordings of respiratory rhythm and local field potentials in the olfactory bulb of freely behaving rats show that each sniff induces early-onset fast gamma oscillation (65–100 Hz) followed by later-onset slow gamma oscillation (40–65 Hz) (Manabe and Mori, 2013). A key point is the time lag between the fast and slow gamma oscillations: the onset of the fast gamma oscillation precedes that of the slow gamma oscillation by about 45 ms on average. Under anesthetized conditions also, odor inhalation induces a shift from fast gamma to slow gamma oscillations similar to that observed in freely behaving rats.

In agreement with the difference in the time window of the fast and slow gamma oscillations, tufted cells and mitral cells differ in their signal timing of odor-induced spike responses. We previously showed in anesthetized rats and mice that odor-inhalation induces early-onset high frequency (about 100 Hz) burst discharges of tufted cells at the middle of inhalation. In contrast, most mitral cells start to respond with low frequency burst discharges (about 45 Hz) at the later phase of the inhalation or at the phase of transition from inhalation to exhalation (Nagayama et al., 2004; Igarashi et al., 2012). The time window of early-onset fast gamma oscillations corresponds to the early-onset response of tufted cells while that of later-onset slow gamma oscillations corresponds to the later-onset response of mitral cells. Based on these observations, we suggest that the sniff-paced early-onset fast gamma oscillations are generated mainly by the tufted cell circuit, while the later-onset slow gamma oscillations are largely due to the mitral cell circuit (Manabe and Mori, 2013) (Figure 3).

How many sister mitral cells and sister tufted cells project their primary dendrites to a single glomerulus? The rabbit olfactory bulb has ~1900 glomeruli, each with a relatively large diameter. Allison and Warwick (Allison and Warwick, 1949) estimated that a single glomerulus in the rabbit olfactory bulb receives primary dendrites from 68 sister tufted cells and 24 sister mitral cells on average. In the mouse olfactory bulb, the size of individual glomeruli is relatively small. A recent study using dye injection into a single glomerulus in the mouse (Kikuta et al., 2013) showed that a single glomerulus is innervated by at least 7 mitral cells, 3 middle tufted cells and 17 juxta-glomerular cells (which include external tufted cells and periglomerular inhibitory neurons). Because dye injection within a single glomerulus may not label all the mitral and tufted cells that innervate the glomerulus, we roughly estimate that a single glomerulus in the mouse olfactory bulb may receive primary dendrites from ~10 sister mitral cells and 20 sister tufted cells.

Thus, the tufted cell circuit of a single glomerular module in mammalian olfactory bulb consists of ~20–68 sister tufted cells and a much larger number of tufted cell-targeted granule cells with intense dendrodendritic synaptic interactions among them in the superficial sub-lamina of the EPL. Because tufted cells extend relatively short lateral dendrites, tufted cells belonging to an activated glomerulus may have small-scale interactions with those tufted cells belonging to nearby co-activated glomeruli and synchronize their activity at fast gamma frequency. We suggest that the tufted cell circuits are the substrate for the sniff-paced early-onset fast gamma oscillations. Tufted cells may send the early-onset signal to focal targets in specific areas of the olfactory peduncle and rostroventral part of the APC with fast gamma synchronization.

The mitral cell circuit of a glomerular module consists of ~10–24 sister mitral cells and numerous mitral cell-targeting granule cells with intense dendrodendritic synaptic interactions in the deeper sub-lamina of the EPL. Because mitral cells project very long lateral dendrites, mitral cells may have large-scale interactions and synchronize at slow gamma frequency with those mitral cells that are distributed over a wide area of the olfactory bulb. The mitral cell circuits may be responsible for generating later-onset slow gamma oscillations. Mitral cells might thus convey later-onset signals dispersedly to nearly all parts of the olfactory cortex with slow gamma synchronization.

The tufted cell circuit and mitral cell circuit can communicate with each other via synaptic and extra-synaptic mechanisms within the glomerulus and also via dendrodendritic synapses of type I granule cells that arborize apical dendrites in both superficial and deep sub-laminas of the EPL (Mori et al., 1983; Orona et al., 1983; Greer, 1987).

Electrical couplings (via gap junctions) between inhibitory interneurons and those between principal cells are involved in the generation of gamma synchronization in the mammalian brain (Galarreta and Hestrin, 2001; Bennett and Zukin, 2004; Hameroff, 2010). Because tufted and mitral cells form gap junctions among themselves within glomeruli and with a subset of periglomerular cells and granule cells (Kosaka and Kosaka, 2005; Kosaka et al., 2005), these gap junctions might be involved in the generation of sniff-paced fast and slow gamma oscillations in the olfactory bulb (Christie et al., 2005; Migliore et al., 2005).

## Tufted cells may provide specificity-projecting circuits, conveying odor information with fast gamma synchronization

The piriform cortex is thought to use spatially distributed overlapping ensembles of active pyramidal cells to represent odors (Neville and Haberly, 2004; Wilson and Sullivan, 2011). Piriform cortex neurons that respond to a given odor are dispersedly distributed across the wide space of the piriform cortex without spatial preference (Litaudon et al., 1997; Illig and Haberly, 2003; Rennaker et al., 2007; Stettler and Axel, 2009; Mitsui et al., 2011). On the other hand, excitatory responses of individual neurons in the piriform cortex are tuned to specific combinations of stimulus odorants (Litaudon et al., 2003; Yoshida and Mori, 2007; Poo and Isaacson, 2011; Wilson and Sullivan, 2011). Therefore, while odor-induced activity is sparsely distributed, odor responses of individual neurons are tuned to specific odorant combinations in the piriform cortex.

An interesting question regarding these apparently opposite properties of the piriform cortex is which of the two afferent axon pathways from the olfactory bulb (tufted cell pathway or mitral cell pathway) mediates each of the properties: “sparse distribution of the odor-induced activity” and “odor tuning of individual neurons.” Pyramidal cells of the APC extend long apical dendrites superficially to layer I and receive afferent axon synaptic inputs from the olfactory bulb at the distal segment of the apical dendrites in layer Ia (APC in Figure 3). In the ventrorostral part of the APC, axons terminating in its layer Ia originate from both mitral cells and tufted cells. In the dorsal and caudal parts of the APC and whole parts of the posterior piriform cortex (PPC), in contrast, almost all afferent axons to layer Ia originate from mitral cells (Haberly and Price, 1977; Neville and Haberly, 2004). Pyramidal cells in the APC also receive inputs from associational axons originating from pyramidal cells of the olfactory peduncle (AON and tenia tecta) and APC itself and terminating on proximal segments of apical dendrites in layer Ib (Ib associational axons, Figure 3).

Poo and Isaacson (2011) have shown that odor tuning of individual neurons in the APC is mediated by Ib associational axon input, rather than direct afferent axon input from the olfactory bulb. Given that individual tufted cells in the olfactory bulb project axons densely to focal targets in areas within the olfactory peduncle (AON and tenia tecta) (Igarashi et al., 2012) (Figure 3), we hypothesize that specific odorant receptor information conveyed by tufted cell axons is synaptically relayed to pyramidal cells in the olfactory peduncle (red P in Figure 3) and then sent to the pyramidal cells of the APC (black P in Figure 3) via Ib associational axons of olfactory peduncle pyramidal cells. In this hypothesis, the odor tuning of individual neurons in the APC is mediated mostly via the tufted cell axon—olfactory peduncle Ib association axon pathways (red lines in Figure 3).

**Figure 5:**
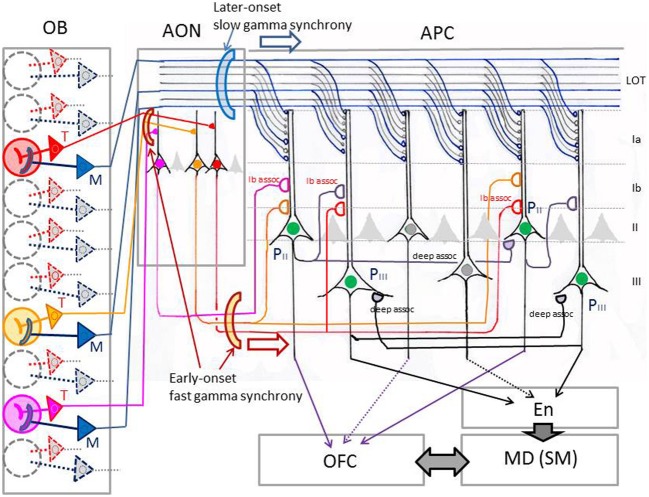
**Schematic diagram of possible functional differentiation between the tufted cell pathway and mitral cell pathway in odor information processing in the neuronal circuits of the piriform cortex.** In this model, red, yellow, and pink glomeruli are assumed to be activated simultaneously by an odor inhalation. Activated tufted cells (T, shown by red, orange, or pink) send the odor information with early-onset fast gamma synchrony to specific target pyramidal cells in the AON, which in turn send the information presumably with fast gamma synchrony to specific-target pyramidal cells in the APC. Activated mitral cells (M, shown by blue) provide dispersedly-projecting feed-forward binding circuits transmitting the spike synchronization timing with later-onset slow gamma synchrony to whole pyramidal cells in the APC. Pyramidal cells in layer II (PII) of the APC project axons directly to the OFC. The layer II pyramidal cells and those in layer III (PIII) of the APC form recurrent association axon synaptic connections (deep assoc. and Ib assoc.) with other pyramidal cells, forming feed-back binding circuits. These pyramidal cells project axons also to the endopiriform nucleus (En), which sends axons to the mediodorsal nucleus (MD) or submedius nucleus (SM) of thalamus. MD and SM provide thalamocortical projections to the OFC, while OFC sends feed-back corticothalamic connections to the MD or SM. LOT, lateral olfactory tract; Ia, Ib, II, and III, layers in the APC. Pyramidal cells with green nucleus in the APC indicate neurons co-activated by an odor inhalation. Recurrent collateral excitatory synaptic connections (deep assoc) among these neurons form feed-back binding circuits.

**Figure 6:**
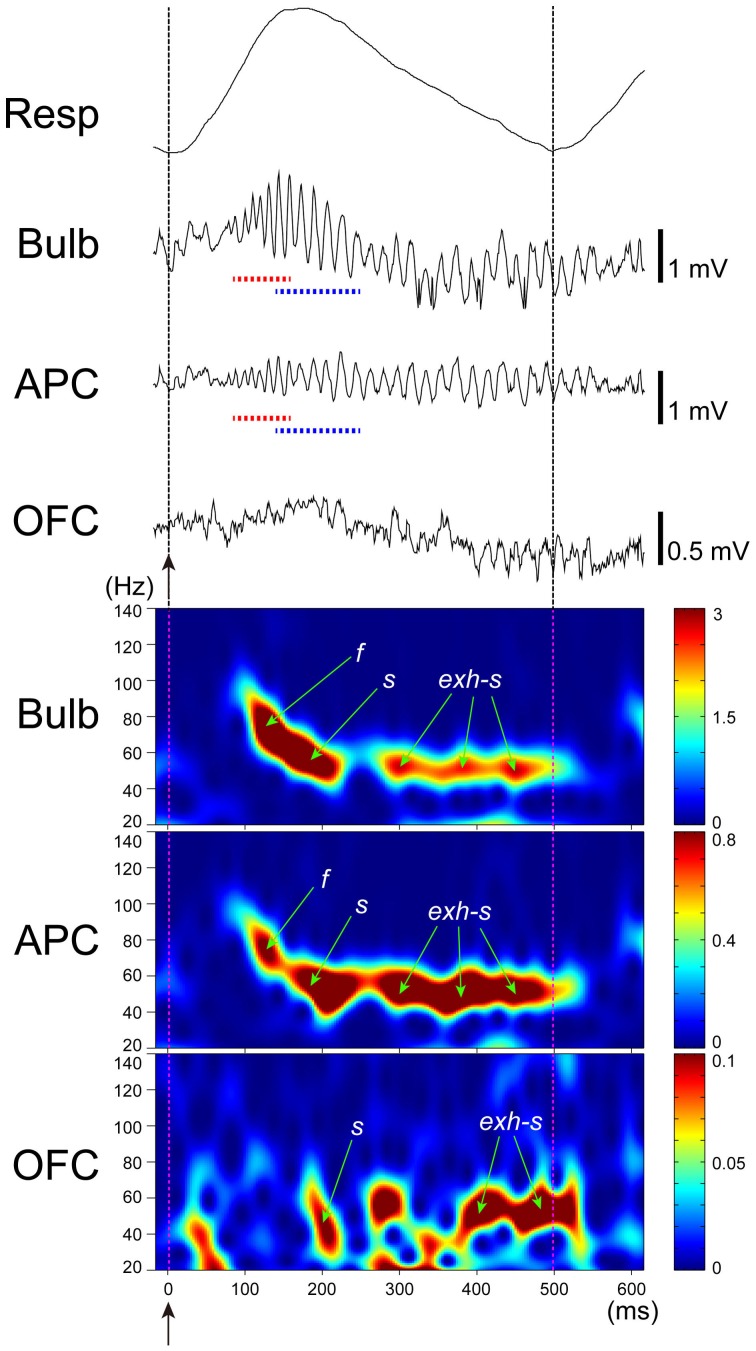
**Gamma oscillation couplings across the olfactory bulb, anterior piriform cortex, and orbitofrontal cortex.** Simultaneous recordings of respiratory pattern (Resp, upward swing indicates inhalation), LFP in the granule cell layer of the olfactory bulb (Bulb), LFP in layer III of the APC (APC), and LFP in the deep layer of OFC (OFC), during micro-arousal. Wavelet analyses of the LFPs are shown in the lower three figures. Broken lines and upward arrows indicate sniff onset. Early-onset fast gamma oscillation is shown by f or red broken line, and later-onset slow gamma oscillation by s or blue broken line. Slow gamma oscillation during the exhalation period is shown by exh-s.

As shown in Figure 6, local field potentials in the APC show sniff rhythm-paced fast and slow gamma oscillations. The early-onset fast gamma oscillations in the APC correspond well in timing and frequency with the early-onset fast gamma oscillations in the olfactory bulb, which are mainly generated by tufted cell circuits. These observations lead us to hypothesize that (1) tufted cells provide specificity-projecting circuits which convey specific odorant receptor signals with early-onset fast gamma synchronization to specific target pyramidal cells in the olfactory peduncle: and that (2) the activated pyramidal cells in the olfactory peduncle then send the signals presumably with fast gamma synchronization to specific target pyramidal cells in the APC via their Ib associational axons (red, orange and pink lines in Figure 5).

Haberly (2001) proposed that the AON, a major area in the olfactory peduncle, detects and stores correlations between “molecular features,” creating representations of particular odorants and odorant mixtures. We speculate that tufted cell signals originating from different glomeruli converge in a specific way onto individual pyramidal cells in the AON, giving rise to the odor tuning of its pyramidal cells (Lei et al., 2006; Kikuta et al., 2008). However, it is not known how the tufted cell signals from different glomerular modules are combined or integrated in individual pyramidal cells in the olfactory peduncle (Brunjes et al., 2011).

We further speculate that Ib associational axon signals from different pyramidal cells in the olfactory peduncle converge under specific rules onto individual pyramidal cells of the piriform cortex, thereby providing the odor-tuning specificity of the pyramidal cells. In fact, pyramidal cells in the pars lateralis of the AON project Ib associational axons to the ventral division of APC (APCv), whereas those in the pars dorsalis of the AON send associational axons to the dorsal division of the APC (APCd) (Haberly and Price, 1978; Luskin and Price, 1983). Future study will detail the connectivity pattern of Ib associational axons of olfactory peduncle pyramidal cells to the pyramidal cells in the APC.

## Mitral cells may provide dispersedly-projecting feed-forward binding circuits, sending the response synchronization timing with slow gamma synchronization

If the odor-tuning specificity of individual pyramidal cells of the APC is determined mainly by the tufted cell axon—olfactory peduncle Ib associational axon pathways, what is the functional role of the direct afferent axon inputs from mitral cells? It has been demonstrated that afferent inputs from a single glomerulus (presumably mitral cell axon inputs) to a pyramidal cell in the APC evoke only weak excitation (Davison and Ehlers, 2011), suggesting that the direct afferent axon inputs from sister mitral cells belonging to a glomerulus may contribute little to the odor-tuning specificity of individual pyramidal cells of the APC.

On the other hand, dye-labeling of physiologically identified mitral cells showed that individual mitral cells project axons dispersedly to nearly all areas of the olfactory cortex, including nearly all parts of the piriform cortex (Figure 3) (Igarashi et al., 2012). Furthermore, sister mitral cells belonging to a given glomerulus project their axons to the piriform cortex in a highly dispersed pattern, with their terminals distributed throughout the piriform cortex (Ghosh et al., 2011; Sosulski et al., 2011). The highly dispersed pattern of mitral cell axon projection to the piriform cortex is independent of the position of the labeled glomerulus in the olfactory bulb (Sosulski et al., 2011), suggesting that sister mitral cells belonging to any glomerulus may project axons to the piriform cortex in a highly dispersed pattern.

Given that mitral cell circuits generate later-onset slow gamma oscillatory activity, we speculate that sister mitral cells belonging to an activated glomerulus provide a mechanism for the dispersion of later-onset slow gamma oscillatory activity across whole parts of the piriform cortex and even across many different areas of the olfactory cortex (Manabe and Mori, 2013). It has been shown that individual target pyramidal cells receive converging inputs from many mitral cells distributed over wide regions of the olfactory bulb (Miyamichi et al., 2011). Because of the widespread projection of lateral dendrites of mitral cells and their intensive dendrodendritic interactions with numerous granule cells, mitral cells belonging to different glomerular modules can synchronize their discharges at the slow gamma range frequency when co-activated (Mori and Takagi, 1977; Kashiwadani et al., 1999). Therefore, mitral cells that are co-activated by odor inhalation would provide later-onset synchronized inputs at slow gamma frequency to pyramidal cells across whole parts of the piriform cortex (Figure 5).

Although input from a single mitral cell axon is weak, nearly simultaneous arrival of synchronized spike inputs from many mitral cell axons may effectively summate their EPSPs in pyramidal cells and thereby strongly modulate pyramidal cell activity in synchrony with the slow gamma oscillatory inputs. We hypothesize that the gamma-synchronized coincident inputs from many mitral cell axons coordinate response timing of pyramidal cells that are spatially distributed across whole parts of the piriform cortex over a sustained time window, providing one of the key elements in “binding” together the spike activities of numerous co-activated pyramidal cells with different tuning specificity (Figure 5).

Recordings of local field potentials in the APC indicate that odor inhalation induces fast gamma oscillations followed by slow gamma oscillations with a time course which closely resembles those observed in the olfactory bulb (Figure 6). This raises the possibility that pyramidal cell activity during the early onset fast gamma oscillations may be due to the tufted axon—Ib associational axon inputs to the pyramidal cells, while that during the later-onset slow gamma oscillations are induced by the combination of preceding tufted cell axon—Ib associational axon inputs and later-onset synchronous inputs from many mitral cells. It is tempting to speculate that the later-onset synchronized inputs from mitral cell axons may cause profound spike synchronization of those pyramidal cells that had been depolarized by preceding early-onset inputs from tufted cell axon—Ib associational axon pathways, while the synchronized inputs may have little influence on those pyramidal cells that had not been depolarized by the tufted cell axon—Ib associational axon inputs. If this is the case, the later-onset diffuse mitral cell inputs might selectively bind the spike activities of only those pyramidal cells that were co-activated via specificity-projecting tufted cell axon—Ib associational axon inputs.

## Recurrent association axon synaptic connections among pyramidal cells of the piriform cortex

Recurrent axon collaterals of pyramidal cells in the piriform cortex form excitatory synaptic connections on dendrites of other pyramidal cells that are distributed widely in the piriform cortex (Figure 5) (Haberly and Presto, 1986; Johnson et al., 2000; Chen et al., 2003; Yang et al., 2004). These recurrent axon collaterals are classified into those that terminate in layer Ib (Ib associational axons) and those that terminate in layers II and III. For example, recurrent axon collaterals of pyramidal cells in the PPC terminate mostly on basal dendrites of other pyramidal cells in layer III, and only a small percentage of them terminate on apical dendrites in layer Ib (Haberly, 2001). We refer to recurrent collaterals that terminate in layer Ib as Ib associational axons and to those that terminate in layers II and III as deep associational axons (Figure 5).

It has been proposed that the Ib and deep associational axon synaptic connections among pyramidal cells in the piriform cortex form networks with iterative recurrent re-excitatory pattern that can store input patterns from the olfactory bulb by plastically changing their synaptic connections (Marr, 1971; Barkai et al., 1994; Haberly, 2001; Neville and Haberly, 2004; Wilson and Sullivan, 2011). A simple model is that during the storage of input patterns the associational synaptic connections would be strengthened between pyramidal cells with different tuning specificity that are co-activated by odor inhalation, while the associational synaptic connections would be weakened between activated and non-activated pyramidal cells. After the learning of the input pattern, the strengthened associational synaptic connections could temporally synchronize the spike activity of those co-activated pyramidal cells with different tuning specificity when the same or similar input patterns arrive from the olfactory bulb (Neville and Haberly, 2004). Computer modeling studies showed that the recurrent associational networks can store and retrieve multiple input patterns that may include olfactory images of numerous different objects (Barkai et al., 1994). The recurrent associational connections among pyramidal cells can thus provide a mechanism for feed-back “binding” of co-activated pyramidal cells based on the memory traces of previously stored input patterns.

As described above, we hypothesize that the synchronous gamma oscillatory inputs from mitral cells cause temporal “binding” of the spike activities of numerous pyramidal cells with different tuning specificity that are co-activated via tufted cell axon—Ib associational axon pathways during odor inhalation. The mitral cell-induced spike synchronization of pyramidal cell activities would facilitate the strengthening of the associational synaptic connections among the co-activated pyramidal cells during the storage of input patterns that are provided by tufted cell—Ib association axon pathways. In summary, we propose a model in which mitral cell pathways provide feed-forward binding circuits, sending the spike synchronization timing to facilitate the storage of olfactory sensory input patterns by causing the spike synchronization of co-activated pyramidal cells at the slow gamma frequency, and thus strengthening association axon synapses among co-activated pyramidal cells with different tuning specificity.

## Gamma oscillation couplings across the olfactory bulb, olfactory cortex, and orbitofrontal cortex

In addition to numerous pyramidal cells, the piriform cortex contains several types of inhibitory interneurons (Neville and Haberly, 2004; Bekkers, 2013). Large horizontal and layer Ia neurogliaform cells in layer I are GABAergic inhibitory neurons that terminate their axons on the distal part of apical dendrites (in layer Ia) of pyramidal cells. Because these Ia inhibitory neurons receive direct synaptic inputs from tufted cell axons or mitral cell axons, they provide feed forward inhibition of pyramidal cells. In the olfactory peduncle areas, fast gamma synchronization of tufted cell activity might be transmitted to pyramidal cells not only by monosynaptic excitatory input from tufted cells but also by di-synaptic inhibitory input via the Ia inhibitory neurons. Similarly, Ia inhibitory neurons in the APC might be used to effectively transmit the slow gamma synchronization of mitral cells via the di-synaptic inhibitory input to pyramidal cells.

Another characteristic type of inhibitory neuron in the olfactory cortex is fast-spiking large multipolar cells that are distributed in layers II and III. Many large multipolar cells are GABAergic basket cells which form basket-like inhibitory terminals around the cell bodies of pyramidal cells. Because the GABAergic basket cells receive deep associational axon inputs from pyramidal cells, they form local feedback inhibitory circuits. This raises the possibility that the local feedback inhibitory circuits form gamma oscillatory sources in the APC and that gamma oscillatory inputs either from tufted cell axon—Ib association axon pathways or mitral cell pathways entrain the gamma oscillations of the APC circuits.

Given that pyramidal cells in layer II of the APC project axons to the OFC, the sniff-paced gamma oscillatory activity of APC pyramidal cells may be transmitted to the OFC. To examine this possibility, we recorded local field potentials simultaneously from the olfactory bulb, APC and OFC (Figure 6). During awake exploratory behavior or awake resting, olfactory bulb and APC typically show sniff-paced early-onset fast gamma oscillation (*f* in Figure 6) followed by later-onset slow gamma oscillation (*s* in Figure 6). In many cases, the early-onset fast gamma oscillation in the APC shows phase-coupling with the early-onset fast gamma oscillation in the olfactory bulb, suggesting a strong functional coupling between the APC and olfactory bulb. Later-onset slow gamma oscillations in the APC also typically show phase-coupling with the later-onset slow gamma oscillations in the olfactory bulb, again suggesting a functional coupling between them.

During awake exploratory behavior and awake resting, the rat OFC occasionally shows sniff-paced fast and slow gamma oscillations with a time course closely resembling those in APC and olfactory bulb (Figure 6). In some sniffs, both fast and slow gamma oscillations occur in the OFC with a similar time course to fast and slow gamma oscillations in the APC, while in other sniffs, the OFC shows only the slow gamma oscillations (Figure 6). These slow gamma oscillations of the OFC sometimes phase-couple with those of APC and olfactory bulb, although the degree of gamma oscillation coupling is weaker than that of the gamma coupling between the APC and olfactory bulb. These results suggest that sniff-paced fast and slow gamma oscillations generated in the olfactory bulb are occasionally transferred to the OFC via the APC.

During awake resting in which rats show a slow respiration pattern with relatively long exhalation phase, the olfactory bulb and APC sometimes show strong coupling of slow gamma oscillation during the long exhalation phase (Manabe and Mori, 2013) (exh-s in Figure 6). This late slow oscillation at the exhalation phase sometimes last for an extended period up to the initial part of the next inhalation phase (Figure 6) and presumably corresponds to the late slow gamma and beta oscillations reported in anesthetized animals (Buonviso et al., 2003; Neville and Haberly, 2003; Cenier et al., 2008). Generation of these late slow oscillations is thought to require mutual interactions between the olfactory bulb and olfactory cortex. We observed that the OFC also shows gamma oscillations that are coupled with those of the APC and olfactory bulb during the long exhalation periods (exh-s in Figure 6), which suggests that gamma oscillation coupling among the olfactory bulb, APC and OFC can occur not only during the inhalation phase, in which the external odor information is transmitted via a bottom-up pathway from the olfactory bulb, but also during the long exhalation period in which the central olfactory system is temporally isolated from the external odor information. This observation raises the possibility that these gamma oscillations can be generated centrally either in the OFC or APC and travel via a top-down pathway to the olfactory bulb. In other words, gamma oscillatory couplings among the olfactory bulb, APC and OFC can be generated either by olfactory sensory inputs or centrally in the brain.

The functional role of this gamma oscillation coupling among the olfactory bulb, APC and OFC during the exhalation-phase remains to be elucidated. It should be noted that rats typically show slow sniffs with a long exhalation phase during eating and that prominent gamma oscillation couplings occur across the olfactory bulb, APC, and orbitofrontal cortex during the long exhalation phase. Because the brain receives retronasal odor stimulation from foods in the mouth during the exhalation phase (Gautam and Verhagen, 2012), these observations raise an interesting possibility that the gamma oscillation couplings are involved in the process of perception of food flavor in the mouth (Shepherd, 2006).

## Behavioral state regulates the generation and coupling of gamma oscillations across the olfactory bulb, piriform cortex, and orbitofrontal cortex

In the neocortex, gamma oscillation and spike synchronization depend on behavioral state and increase with arousal, attention, and expectancy (Herculano-Houzel et al., 1999; Fries, 2009). They are absent or greatly diminished during sleep states. Similarly, conscious awareness of olfactory sensory information of the external world depends on behavioral state, occurring during alert wakefulness but absent during sleep states. Several studies have pointed out the relationship between gamma oscillations in the olfactory cortex and behavioral states (Freeman, 1975; Kay, 2003). We therefore addressed the question of whether behavioral state regulates the generation of sniff-paced fast and slow gamma oscillations in the olfactory bulb, and found that the sniff-paced gamma oscillations occur throughout the waking states, but disappear during slow-wave sleep and REM-sleep (Manabe and Mori, 2013). During the shallower stage of slow-wave sleep, rats sometimes show micro-arousals for a short period, and the sniff-paced fast and slow gamma oscillations occur only during these short periods of micro-arousal. At the end of micro-arousal, the neocortical EEG resumes the slow-wave sleep pattern, and the sniff-paced gamma oscillations disappear. These results indicate that gamma oscillation generation in the olfactory bulb is under the control of behavioral state, particularly the awake and sleep states. In other words, activated states of olfactory bulb are required, in addition to odor inhalation, to generate gamma-band synchrony of tufted cells and mitral cells. Furthermore, it has been shown that task demands enhance gamma oscillations in the olfactory bulb (Beshel et al., 2007).

Neuronal mechanisms for the behavioral state dependency of gamma oscillation generation are not well-understood. Because mitral/tufted cells and granule cells in the olfactory bulb are under the control of centrifugal neuromodulatory systems (cholinergic, noradrenergic, and serotonergic systems) which change their activity with behavioral state, these neuromodulatory systems are candidates for the mediation of behavioral state dependency. For example, a behavioral state-dependent global change in cholinergic tone modulates granule-to-mitral/tufted dendrodendritic synaptic inhibition and thus modulates gamma oscillation generation (Tsuno et al., 2008).

Behavioral state also regulates the generation of sniff-paced fast and slow gamma oscillations in the APC (Onisawa et al., unpublished). The fast and slow gamma oscillations are frequently observed in the APC during awake behaving periods, but are almost completely absent during slow-wave sleep and REM sleep. Recurrent inhibitory circuits between pyramidal cells and GABAergic basket cells are thought to be responsible for gamma oscillation generation in the APC, and are also under the control of the neuromodulatory system (Gellman and Aghajanian, 1993; Barkai and Hasselmo, 1994; Hasselmo and Barkai, 1995). In addition, state-dependent sensory gating in the olfactory cortex is regulated by strong neuromodulatory input from the brainstem reticular activation system, which includes areas surrounding the pedunculo-pontine tegmental nuclei (Murakami et al., 2005).

In the OFC also, sniff-paced gamma oscillations are regulated by behavioral state, being occasionally present during wakefulness but absent during sleep. Furthermore, the coupling of sniff-paced gamma oscillations among the olfactory bulb, APC and OFC occurs occasionally during awake periods, but is absent during sleep periods. This suggests that external odor information can be transmitted with gamma oscillation from the olfactory bulb through the APC to the OFC during wakefulness, but that transmission is completely shut down or greatly reduced during sleep. The neuronal circuit mechanism for the generation of gamma oscillatory activity in the OFC is unknown. Because optogenetic activation of fast-spiking inhibitory interneurons induces gamma oscillations in the neocortex (Cardin et al., 2009), we speculate that fast-spiking inhibitory interneurons may participate in the generation of sniff-paced gamma oscillatory activity in the OFC. However, virtually nothing is known about local neuronal circuits in the OFC.

An intriguing question for future research is whether the attention and expectancy that accompany cholinergic activation modulate gamma oscillation coupling across the olfactory bulb, APC and OFC in such a way that odor information transfer to the OFC is greatly facilitated when animals are paying attention to the odor information or expecting an odor-associated reward.

## Possible functional roles of gamma oscillation couplings in conscious olfactory perception

In summary, we propose a model of the neuronal circuit mechanism of odor information transfer via gamma oscillation couplings across the olfactory bulb, APC and OFC. In this model, the spatial arrangement of glomerular modules in the mouse olfactory bulb can be viewed as a map of the ~1000 types of odorant receptor-specific gamma oscillators. To perceive an odor, brain needs to know the input pattern from these gamma oscillators; i.e., the specific combination of gamma oscillators co-activated by the odor inhalation. Each glomerular module contains two types of gamma oscillatory source with different onset latencies, tufted cell circuit, and mitral cell circuit. We propose that the sniff-paced early-onset fast gamma oscillations are mainly generated by tufted cell circuits, whereas later-onset slow gamma oscillations are mainly due to mitral cell circuits. Sniff-paced fast and slow gamma oscillations also occur in the APC with similar onset time lag. The early-onset fast gamma oscillations in the APC often show phase-coupling with early-onset fast gamma oscillations in the olfactory bulb, suggesting functional coupling between tufted cell inputs and fast gamma oscillation in the APC. The later-onset slow gamma oscillations in the APC occur in synchrony with the later-onset slow gamma oscillations in the bulb, suggesting that afferent axon inputs from mitral cells are responsible for the later-onset slow gamma oscillations in the APC.

We propose the hypothesis that tufted cell circuits and mitral cell circuits play distinct functional roles in odor information processing and memory formation in neuronal circuits of the piriform cortex. Tufted cells may provide specificity-projecting circuits which send specific odor information to focal targets during the time window of early-onset fast gamma synchronization, while mitral cells give rise to dispersedly-projecting feed-forward binding circuits, transmitting the response synchronization timing with later-onset slow gamma synchronization to all parts of the piriform cortex. Our model suggests a sequence of bindings in the piriform cortex: a small-scale binding by early-phase fast gamma synchrony of tufted cell inputs followed by a larger-scale binding by later-onset slow gamma synchrony of mitral cell inputs. One possible scenario is that the later-onset slow-gamma synchrony of mitral cell inputs cause spike synchronization of the large ensemble of piriform cortex pyramidal cells that had been co-activated by the preceding early-onset fast-gamma synchrony of tufted cell axon—Ib associational fiber inputs. The larger-scale feed-forward binding by later-onset mitral cell inputs may induce selective “binding” of the spike activity of co-activated pyramidal cells with different odor-tuning specificity, causing plastic changes in the recurrent associational synaptic connections among them. The large-scale feed-forward binding by mitral cell inputs and the large-scale feed-back binding by the recurrent associational synaptic connections may work together both for the memorization and retrieval of odor images of objects. If the groupings of pyramidal cell activities are associated with reward or punishment, the temporal binding of activities of the large ensemble of pyramidal cells may lead to either appetitive behavior or aversive behavior (Choi et al., 2011).

**Figure 7:**
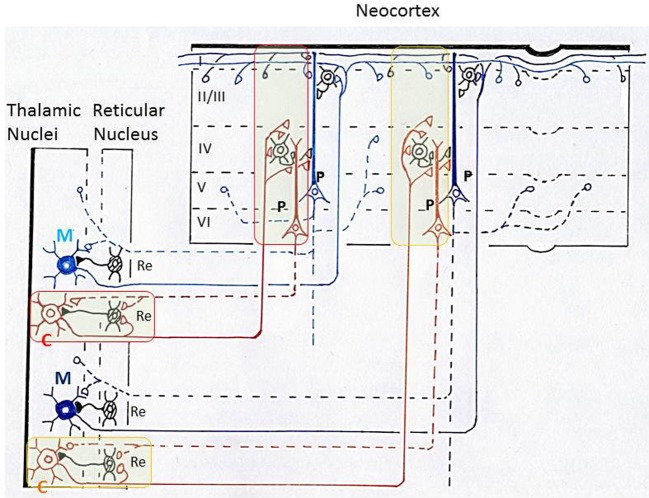
**Schematic diagram illustrating thalamo-cortico-thalamic networks and the “matrix and core theory” of Jones.** This diagram is based on Llinas et al. (1998) and Jones (2001). Core neurons (C) in the thalamus form specificity-projecting circuits (shown by brown lines and surrounded by red or yellow lines) that involve specific cortico-thalamic connections from layer VI pyramidal cells. Matrix neurons (M) in the thalamus give rise to non-specific binding circuits (shown by blue lines) that involve diffuse projections from layer V pyramidal cells. Jones proposed that the above two types of thalamo-cortico-thalamic networks form a substrate for synchronization of widespread populations of neurons in the thalamus and cortex during high-frequency oscillations that underlie discrete conscious events (Jones, 2009). P, pyramidal cells in the neocortex; Re, inhibitory neurons in the reticular nucleus of thalamus; I, II/III, IV, V, VI, layers in the neocortex.

Conscious perception of visual, auditory, somatosensory, and gustatory senses is thought to depend on thalamocortical circuits. A basic consensus in the studies of neuronal mechanism of consciousness is the need to rapidly integrate and bind information that is situated across distinct cortical and thalamic regions (Llinas et al., 1998; Baars, 2002; Crick and Koch, 2005). A large ensemble of widespread populations of neurons in the thalamus and neocortex shows synchronous oscillatory activities at the gamma frequency range during conscious cognitive processes (McCormick and Bal, 1997; Steriade, 2000). It has been proposed that large-scale gamma oscillation couplings of activities across many thalamo-cortical and cortico-thalamic networks generate the functional states that characterize conscious cognitive processes (Figure 7) (Llinas et al., 1998).

In exploring the function of these thalamo-cortical networks, Jones (2001, 2009) classified thalamo-cortical projection neurons into two types, core neurons and matrix neurons, and proposed that they provide a basis for the gamma oscillation couplings in the thalamus and neocortex. Core neurons project axons in a topographically ordered fashion to middle layers of the neocortex in an area-specific manner, forming a basis for the relay of place- and modality-specific information to the neocortex. In striking contrast, matrix neurons project axons diffusely to superficial layers of the neocortex over wide areas, unconstrained by boundaries between areas, forming a basis for the dispersion of activity into the thalamocortical network across large areas of neocortex (Figure 7).

It should be underscored that the possible functional differentiation between tufted and mitral cells in the olfactory bulb closely resembles that between core and matrix neurons in the thalamus: tufted cells and core neurons may provide specificity-projecting circuits, whereas mitral cells and matrix neurons may provide dispersedly-projecting binding circuits. Both the thalamus and olfactory bulb have parallel pathways with a distinct axonal projection pattern. These results raise the possibility that the thalamus and olfactory bulb may use a similar neuronal circuit logic of parallel pathways for the transfer of sensory signals to the cortex and for the binding of widely distributed cortical neuron activity with small-scale and large-scale gamma synchronizations. Such neuronal circuit logic, together with the dispersed axonal projection, might provide a basis for generating large-scale synchronized activities of cortico-cortical or thalamocortical networks that underlie the conscious perception of objects.

We suggest that generation of olfactory consciousness requires neuronal circuit mechanisms for the binding of distributed neuronal activities in which each constituent neuron might represents a specific component of an olfactory percept. Besides the olfactory bulb, piriform cortex and OFC, sniff rhythm-paced gamma oscillations are observed in the olfactory tubercle, cortical amygdaloid nuclei, lateral entorhinal cortex, and medial prefrontal cortex. A major challenge in future studies of the neuronal circuit mechanism of olfactory consciousness is therefore to elucidate the basic logic of large-scale gamma oscillation couplings and the “binding” of activities of widespread populations of neurons over many different areas of the olfactory cortex, as well as across the olfactory cortex, prefrontal cortex, thalamus and basal ganglia. Couplings of odor-induced and centrally-generated synchronized oscillatory activities across large-scale networks in the central olfactory system might also be a basic strategy for the transfer of cognitive information of odor objects into neuronal circuits for the planning of behavioral responses.

### Conflict of interest statement

The authors declare that the research was conducted in the absence of any commercial or financial relationships that could be construed as a potential conflict of interest.
